# Seasonal Patterns of Gastrointestinal Illness and Streamflow along the Ohio River

**DOI:** 10.3390/ijerph9051771

**Published:** 2012-05-07

**Authors:** Jyotsna S. Jagai, Jeffrey K. Griffiths, Paul K. Kirshen, Patrick Webb, Elena N. Naumova

**Affiliations:** 1 U.S. Environmental Protection Agency, Research Triangle Park, NC 27711, USA; 2 Tufts University School of Medicine, Boston, MA 02111, USA; Email: jeffrey.griffiths@tufts.edu (J.K.G.); elena.naumova@tufts.edu (E.N.N.); 3 Institute for the Study of Earth, Oceans, and Space, University of New Hampshire, Durham, NH 03824, USA; Email: paul.kirshen@unh.edu; 4 Friedman School of Nutrition Science and Policy, Tufts University, Boston, MA 02111, USA; Email: patrick.webb@tufts.edu; 5 Tufts University School of Engineering, Medford, MA 02155, USA

**Keywords:** drinking water quality, gastrointestinal infections, hydrology, pathogens, seasonality, streamflow

## Abstract

Waterborne gastrointestinal (GI) illnesses demonstrate seasonal increases associated with water quality and meteorological characteristics. However, few studies have been conducted on the association of hydrological parameters, such as streamflow, and seasonality of GI illnesses. Streamflow is correlated with biological contamination and can be used as proxy for drinking water contamination. We compare seasonal patterns of GI illnesses in the elderly (65 years and older) along the Ohio River for a 14-year period (1991–2004) to seasonal patterns of streamflow. Focusing on six counties in close proximity to the river, we compiled weekly time series of hospitalizations for GI illnesses and streamflow data. Seasonal patterns were explored using Poisson annual harmonic regression with and without adjustment for streamflow. GI illnesses demonstrated significant seasonal patterns with peak timing preceding peak timing of streamflow for all six counties. Seasonal patterns of illness remain consistent after adjusting for streamflow. This study found that the time of peak GI illness precedes the peak of streamflow, suggesting either an indirect relationship or a more direct path whereby pathogens enter water supplies prior to the peak in streamflow. Such findings call for interdisciplinary research to better understand associations among streamflow, pathogen loading, and rates of gastrointestinal illnesses.

## 1. Introduction

Waterborne pathogens are a significant health concern worldwide, with an estimated 88% of diarrheal diseases attributable to unsafe water supplies [1]. These food and waterborne pathogens are a concern even in the United States, with approximately 76 million cases of illness and 5,000 deaths caused by gastrointestinal infections annually [2]. A recent assessment of waterborne disease outbreaks in the U.S. demonstrated that 780 of the 833 outbreaks that occurred between 1971 and 2006 were associated with drinking water and of these 87.2% occurred in public water supplies [3]. 

Disease causing waterborne protozoa, such as *Giardia* and *Cryptosporidium* spp., are ubiquitous in water supplies and have been detected in both surface water sources and finished, post-treatment, drinking water supplies [4,5,6]. These protozoa were detected in 97% of samples of raw surface waters tested in Eastern and Midwestern states in the United States [5] and *Cryptosporidium oocysts* were detected in 34 of 35 samples of river waters in Washington State [7]. These pathogens are not only common in the environment, the concentration of the pathogens demonstrate seasonal patterns in surface waters [7,8,9,10].

The waterborne diseases caused by these protozoa in humans also demonstrate seasonal patterns in incidence. Increases in cryptosporidiosis incidence are seen during the warm, rainy season in tropical climates [11,12,13]. In temperate climates, increases in incidence of cryptosporidiosis are seen in the spring and fall in [14,15,16,17]. Fewer studies have explored the seasonal patterns in incidence of giardiasis; however similar patterns have been seen. In the tropical climate of southern India, giardiasis demonstrated higher rates in months with higher rainfall [18] while the arid climate of Lebanon, giardiasis demonstrated consistent rates throughout the year with little seasonal variation [19]. 

Increase in diarrheal disease incidence has been shown to be associated with water quality parameters, such as turbidity [20,21,22,23]. However, limited studies have been conducted on the association between rates of gastrointestinal illness and streamflow, or river discharge, which is highly correlated with turbidity (|r| > 0.4; *p* < 0.01) [24]. A study conducted in England and Wales demonstrated that the incidence rate of cryptosporidiosis was positively related to the maximum average streamflow in that month for months between April and July. Between August and November, the cryptosporidiosis incidence rate was also positively associated with maximum river flows but only after adjusting for the previous month’s temperature, rainfall, and cryptosporidiosis rate [25]. 

Environmental conditions can affect the distribution of these protozoa, and other pathogens, in surface drinking water supplies. For example, *Cryptosporidium* oocysts are transmitted via the feces of infected animals, primarily cattle. The manure may be spread on land where the oocysts may survive for over a year [26] and may be transferred into rivers waters under the correct conditions, such as being within the 100 year flood plain and including a large enough farm area [27]. Sewage effluent has also been recognized as a source of pathogen contamination of surface waters [27,28]. Once the pathogens are washed into rivers, specific climatic and soil conditions allow them to survive for extended periods of time and be re-suspended at a later time. 

Each river has unique hydrological characteristics, such as streamflow and water temperature, which may affect the rates and seasonal patterns of waterborne diseases in communities that rely on drinking water from that particular river. While most pathogens are removed from drinking water supplies in the treatment process through coagulation, sedimentation, and filtration [29] some pathogens, such as *Cryptosporidium*, are resistant to the disinfectants typically used in drinking water treatment [30]. In general, water treatment only reduces pathogen loads rather than completely removing pathogens and higher pathogen concentrations are expected in the spring time with increased runoff and snowmelt. A study of gastrointestinal outbreaks between 1948 and 1994 in the U.S. demonstrated that 51% of outbreaks were preceded by heavy rainfall events [31]. This demonstrates that treatment facilities can be overburdened by upstream sewage discharges or increases in streamflow due to rainfall. Streamflow, a commonly monitored measure, is highly correlated with biological contamination [24], and therefore can be used as a proxy for drinking water contamination for which monitoring data is not systematically maintained.

**Figure 1:**
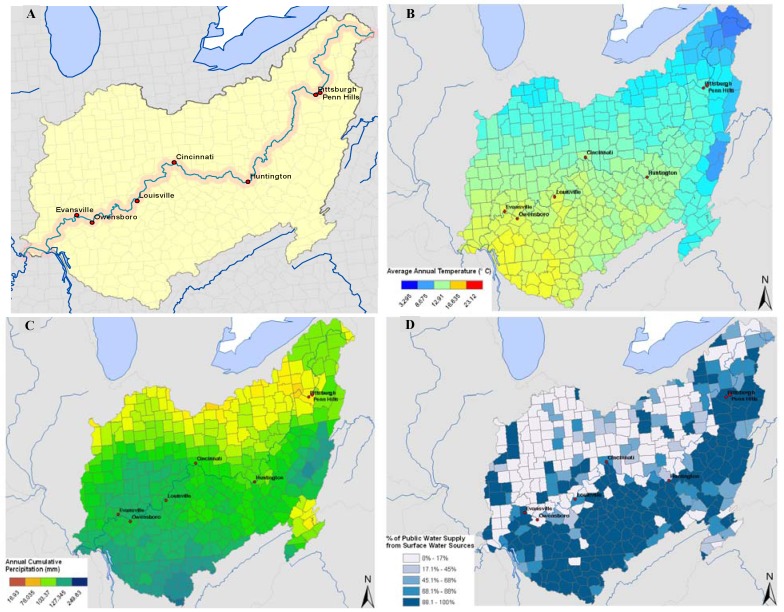
Selected cities and characteristics of the Ohio River watershed. Cities (populations >50,000 persons) which fall within a 10-mile buffer of the Ohio River (Panel A). Average annual temperature (°C) by county (Panel B) and annual cumulative precipitation (mm) by county in the Ohio River watershed (PRISM Data - http://www.prism.oregonstate.edu/) (Panel C). Percent of county public water supply from surface water sources in the Ohio River watershed (Panel D) (USGS Data, http://water.usgs.gov/watuse/data/2000/index.html).

In this study, we assessed seasonal patterns for gastrointestinal illness in the elderly for counties along the Ohio River and compared them to the seasonal patterns of streamflow. The elderly are a vulnerable subpopulation for gastrointestinal infections. They often have cardiac, renal, or other illnesses which affect their ability to compensate for the fluid shifts seen with gastroenteritis and rates of hospitalization for gastroenteritis rise with age [32]. The Ohio River was selected as a case study because it serves as a source of drinking water for approximately five million people in 29 public drinking water utilities [33]. The most recent assessment of water quality along the Ohio River demonstrated that the entire river fully supports public water supply [33]. However based on the Clean Water Act Standards, several areas were listed on the 303(d) impairment list due to violations of criteria for iron, temperature, and dissolved oxygen [33]. In fact, two-thirds of the river was listed as impaired for contact recreation due to the presence of bacteria [33]. Additionally, the Ohio River is fairly homogenous in meteorological characteristics, such as temperature and precipitation (Figure 1).

## 2. Methods

### 2.1. Location Selection

Cities in close proximity to the Ohio River were selected for this analysis using ArcGIS 9.1 (ESRI, Redlands, CA, USA). A 10-mile buffer was created along the river and overlaid with a layer of cities with populations greater than 50,000 people. Those cities which fell within the river buffer were selected for analysis and we analyzed data for the counties in which each of these cities fell. Figure 1 shows the 7 cities from 6 counties in the Ohio River watershed that were used for the analysis along with annual average temperature, annual cumulative precipitation, and the proportion of public water supplied by surface water among these communities. 

### 2.2. Outcome Data

Hospitalization records for persons aged ≥65 years were abstracted for each of the six selected counties for a 14-year period (1 January 1991–31 December 2004) from the Centers for Medicare and Medicaid Services (CMS). About 96% of all adults aged ≥65 years are CMS beneficiaries, therefore their hospitalization charges are reflected in this dataset [34,35]. Each hospitalization record contains individual patient information including state of residence, sex, age at admission, dates of admission and discharge, and ten ICD-9-CM system diagnosis codes. For this analysis, we considered records with the following diagnoses in any of these ten diagnosis codes: cryptosporidiosis (ICD 007.2, 007.4) [36], giardiasis (ICD 007.1), other protozoa (ICD 007.8, 007.9), all protozoa (ICD 007.1, 007.2, 007.4, 007.8, 007.9), viral GI (ICD 008.6), ill-defined GI infections (ICD 008.5, 008.8, 009), GI symptoms (ICD 558.9, 787) and all GI infections without Clostridium difficile (ICD 001-009 excluding 008.45). We removed *Clostridium difficle* from this outcome as it is primarily a nosocomial infection. Hospitalization records were aggregated according to each patient’s diagnosis code, location of residence, and date of admission. Annual rates for each outcome were calculated for each county using the linearly interpolated elderly population for 1997 (midpoint of data timeframe) from 1990 and 2000 U.S. Census Data as the denominator. We also created a weekly time series of rates for each county for each outcome of interest for seasonality assessment. 

### 2.3. Exposure Data

We abstracted daily streamflow data for each of the six counties selected along the Ohio River, as described above, from publicly available U.S. Geological Survey (USGS) 37] databases for the study period (1991–2004). In cases when more than one monitoring station provided data for the entire study period the station which was closest to the main river stem (Ohio River), based on latitude and longitude coordinates of the station, was chosen. The daily streamflow data was aggregated on a weekly level. We did not have any missing data and therefore, no interpolation was needed.

### 2.4. Exploratory Assessment of Correlation

Spearman cross-correlations were calculated for weekly streamflow between the six selected counties to assess similarities in seasonal pattern of streamflow along the river. Spearman cross-correlations were also calculated between weekly outcome rates and streamflow for each selected county along the Ohio River for outcomes with sufficient disease outcome counts; ill-defined GI infections, GI symptoms, and all GI infections. We considered time-lagged correlations with weekly GI disease outcome rates lagged by a week after peak streamflow for up to twenty four weeks, 6 months, to assess associations with the nadir or seasonal minimum in outcome. We also considered the correlation between weekly outcome rate and streamflow for the previous week, two weeks previous, three weeks previous, *etc*. up to twenty five weeks previous. 

### 2.5. Seasonality Assessment

We assessed seasonal patterns for each outcome and each county selected along the Ohio River using Poisson harmonic regression. Seasonality is characterized as systematic, periodic fluctuations within the course of a year. It is assessed by several parameters: (1) the time when the seasonal curve reaches its maximum; (2) annual maximum value (peak); and (3) annual minimum value (nadir) [16]. These seasonal parameters are calculated based on values predicted by the harmonic regression (Equation 1):


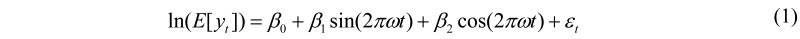


where *y_t_* is a time-series of rates for a specific outcome, *t* is time in weeks, *ω* is frequency (*ω =* 1/52.25), *β*_0_ is intercept, *β*_1_, and *β*_2_ are regression parameters, and *ε_t_* is the error term. The relative intensity, a measure of the shape of the seasonal pattern from peak to nadir, is calculated by dividing the estimated seasonal maximum value by the estimated seasonal minimum value. 

This model was used to assess the seasonal peak timing of streamflow, and the seasonal peak timing of each outcome, GI symptoms, ill-defined GI infections and all GI infections. The other outcomes of interest which we had defined had a low number of reported counts by county, with many weeks of zero counts, for which seasonality cannot be assessed using Poisson harmonic regression. Based on exploratory data analysis, we found that each time series, streamflow and all outcomes, demonstrated only one seasonal periodic cycle therefore a single harmonic term was used for seasonality assessment. 

Seasonality was also assessed adjusting for weekly average streamflow by county using the Poisson harmonic regression equation (Equation 2): 


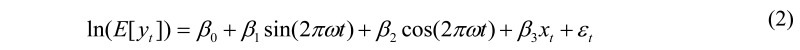


In this case–*x_t_* is the weekly time series for average streamflow for a given location. Adjusting for streamflow allows us to take into account any seasonal variation which may be due to streamflow and only assess the seasonality of the outcome. The results of the models are presented in terms of peak timing with confidence intervals, estimated from regression parameters and relative intensity, or amplitude. Details of the derivation of these seasonal curve properties predicted by the Poisson harmonic regression are given in the supplemental material (Supplemental Material: Figure A). 

## 3. Results

We selected six counties with population centers with more than 50,000 residents within a 10-mile buffer of the Ohio River (Figure 1) and compared the seasonal patterns for rates of hospitalization for gastrointestinal infections with the seasonal patterns in streamflow in the selected counties. The streamflow data for Jefferson Co, KY and Daviess Co, KY were collected from Ohio River monitoring stations, which reported higher values for streamflow since it is a larger water body, whereas data for the other counties were gathered from tributaries which flowed into the Ohio River. Regardless of where the streamflow data were collected, the variability is similar as suggested by the coefficent of variation which was close to one for all sites (Table 1). 

**Table 1:** Descriptive statistics for streamflow (ft^3^/sec). Weekly mean, standard deviation, minimum, maximum and interquartile range over the 14-year period 1991–2004. Counties are listed in order based on the flow of the river, east to west.

	STREAMFLOW (FT^3^/SEC)					
	MEAN	STD. DEV	MIN	MAX	INTERQUARTILE RANGE	COEFFICENT OF VARIATION
Allegheny Co, PA	19,588.48	15,728.91	2,431.43	80,185.71	6,349.29–28,803.57	0.803
Cabell Co, WV	50.93	82.47	0.22	780.86	4.72–65.21	1.619
Hamilton Co, OH	1,419.22	1,684.33	81.00	12,822.86	343.64–1,785.93	1.187
Jefferson Co, KY	124,019.44	108,094.86	8,595.71	620,571.43	37,382.14–183,607.14	0.872
Daviess Co, KY	135,325.20	112,379.97	9,528.57	627,142.86	45,875.00–193,714.29	0.830
Vanderburgh Co, IN	31,707.29	30,406.60	2,998.57	205,857.14	10,685.71–42,600.00	0.959

Weekly streamflow between the six selected counties is strongly correlated suggesting that the time series for streamflow demonstrate a similar pattern in each county (Supplemental Material: Table A). This analysis allowed us to confirm that regardless of where the data were collected (on the Ohio River or a tributary) the seasonal patterns in streamflow remain consistent. 

Annual rates were calculated for each outcome of interest (Table 2). As documented elsewhere, rates for specific disease outcomes, such as cryptosporidiosis and giardiasis, were low because hospitalization and testing for specific pathogens is limited and under-reporting of these infections occurs [36]. Disease counts and annual rates were highest for GI symptoms, ill-defined GI infections, and all GI infections; thus, these three outcomes were used for correlation and seasonality assessment. 

**Table 2:** Total cases and annual rate (per 10,000) for each outcome and for each selected county in the Ohio River Watershed over the 14-year period 1991–2004. Counties are listed in order based on the flow of the river, east to west. Those outcomes in bold provided sufficient counts for seasonality assessment.

COUNTY NAME	ELDERLY POPULATION (65 + Y.O.)	CRYPTO-SPORIDIOSIS *		GIARDIASIS *		OTHER PROTOZOA *		VIRUSES *		GI SYMPTOMS *		ILL DEFINED GI INFECTIONS *		ALL GI INFECTIONS *	
		CASES	RATE	CASES	RATE	CASES	RATE	CASES	RATE	CASES	RATE	CASES	RATE	CASES	RATE
Allegheny Co, PA	229,290	1	0.00	34	0.11	2	0.01	57	0.18	41,024	127.80	3,156	9.83	4,873	15.18
Cabell Co, WV	15,586	1	0.05	1	0.05	1	0.05	3	0.01	4,879	223.60	291	13.34	392	17.96
Hamilton Co, OH	114,280	1	0.01	22	0.14	1	0.01	15	0.05	26,264	164.16	1,242	7.76	1,850	11.56
Jefferson Co, KY	92,850	0	0.00	9	0.07	1	0.01	7	0.02	24,082	185.26	1,503	11.56	2,016	15.51
Daviess Co, KY	12,283	0	0.00	0	0.00	0	0.00	1	0.00	3,818	222.03	185	10.76	236	13.72
Vanderburgh Co, IN	26,233	0	0.00	2	0.05	2	0.05	10	0.03	7,002	190.65	364	9.91	527	14.35
Total (all counties)	490,522	3	0.00	68	0.10	7	0.01	93	0.14	107,069	155.91	6741	9.82	9,894	14.41

* ICD 9-CM code used for each outcome: cryptosporidiosis (ICD 007.4, 007.2); giardiasis (ICD 007.1); other protozoa (ICD 007.8, 007.9); viruses (ICD 008.6); GI symptoms (ICD 558.9, 787); ill-defined GI infections (ICD 008.5, 008.8, 009); all GI infections (ICD 001-009 W/O 008.45).

**Figure 2:**
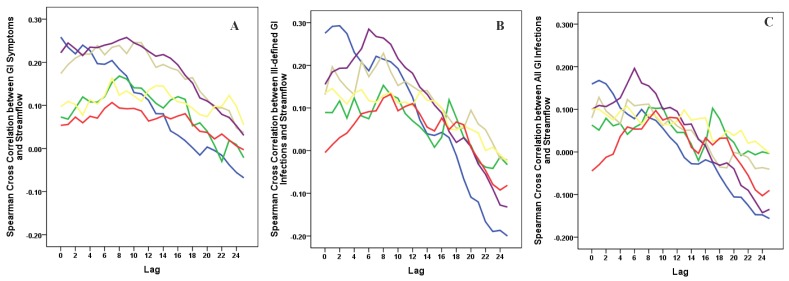
Cross correlations between streamflow and outcomes rates for GI symptoms (Panel A), Ill-defined GI infections (Panel B), and All GI infections (Panel C) with lags up to 25 weeks for each of the six selected counties. (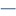 Allegheny Co, PA, 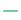 Cabell Co, WV, 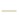 Hamilton Co, OH, 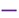 Jefferson Co, KY, 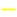 Daviess Co, KY, 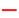 Vanderburgh Co, IN).

Cross-correlations between streamflow and all three outcomes, GI symptoms, ill-defined GI infections, and all GI infections, for each county revealed that in general, high correlations between these GI outcomes and streamflow were synchronous. With increasing lag periods the correlation weakened (Figure 2). All counties, except Cabell Co, WV and Vanderburgh Co, IN, demonstrated significant synchronous (Lag0) correlation values for GI symptoms and ill-defined GI infections. These correlations remained significant when considering lagged relationships up to twelve weeks. All GI infections exhibited weaker associations and the correlations were only significant in Allegheny Co, PA, Jefferson Co, KY and Daviess Co, KY. For all three outcomes of interest the highest correlations were seen between 6 and 12 week lags. At a lag of 6 months, 24–25 weeks, the correlation becomes negative suggesting that the seasonal pattern for gastrointestinal infections and streamflow are similar with peaks and nadirs occurring at roughly the same time. 

Seasonality assessment estimated the peak timing for streamflow between the 9th (~4th week of February) and 13th week (~4th week in March) for all selected counties and the relative intensity of the seasonal curve ranges from 3.26 to 10.18 (Table 3). The highest relative intensity of 10.18 was estimated for Cabell County, WV indicating that streamflow at this site has a greater difference between minimum and maximum values. GI symptoms demonstrated a significant seasonal pattern in counties in the Ohio River watershed except Vanderburgh Co, IN (Table 4). Ill-defined GI infections (Table 5) and all GI infections (Table 6) demonstrated significant seasonal patterns in all counties except Hamilton Co, OH. The peak timing for all three disease outcomes was seen in the early part of the year, ranging from mid January to early March. For GI symptoms, the latest peak timing, ~1st week of March, was estimated for Allegheny Co, PA which is the upper-most site on the river. For most counties, the peak timing of disease rate preceded the peak timing in streamflow. Along the Ohio River, for all three outcomes of interest, adjustment for streamflow did not affect the point estimates of peak timing (Table 4–Table 6). With adjustment for streamflow, the point estimates for peak timing of all three disease outcomes remains within a week of the original estimate. 

**Table 3:** Seasonal pattern for streamflow over 14 year-period for counties along the Ohio River with estimated peak timing, 95% confidence interval, relative intensity, and source of discharge data (main river stem or tributary). Counties are listed in order based on the flow of the river, east to west. Seasonal parameters which are significant (*p* < 0.05) are denoted with an asterisk (*).

COUNTY AND SEASONAL PATTERN	PEAK ESTIMATE (95% CI)	RELATIVE INTENSITY	DATA SOURCE
ALLEGHENY CO, PA	8.795 (8.793, 8.797) * ~4th week of February	3.56	Allegheny River (Tributary)
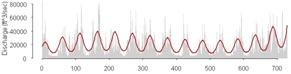			
CABELL CO, WV	9.758 (9.724, 9.792) * ~1st week of March	10.18	East Fork Twelvepole Creek (Tributary)
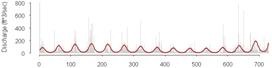			
HAMILTON CO, OH	12.404 (12.402, 12.405) * ~3rd week of March	4.88	Little Miami River (Tributary)
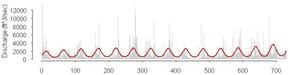			
JEFFERSON CO, KY	10.257 (10.256, 10.257) * ~1st week of March	5.27	Ohio River (Main Stem)
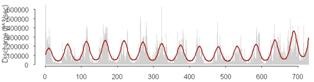			
DAVIESS CO, KY	10.480 (10.480, 10.481) * ~1st week of March	4.83	Ohio River (Main Stem)
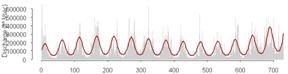			
VANDERBURGH CO, IN	13.088 (13.088, 13.089) * ~4th week of March	3.26	Wabash River (Tributary)
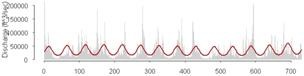			

**Table 4:** Seasonal pattern for GI symptoms over 14 year-period for counties along the Ohio River with estimated peak timing and relative intensity both with and without adjustment for streamflow. Counties are listed in order based on the flow of the river, east to west. Significant (*p* < 0.05) seasonal peak estimates are denoted with an asterisk (*).

	WITHOUT ADJUSTMENT		ADJUSTING FOR STREAMFLOW ^†^	
COUNTY AND SEASONAL PATTERN	PEAK ESTIMATE (95% CI)	RELATIVE INTENSITY	PEAK ESTIMATE (95% CI)	RELATIVE INTENSITY
ALLEGHENY CO, PA	10.13 (9.95, 10.31) * ~1st week of March	1.19	10.28 (8.01, 12.54) * ~1st week of March	1.17
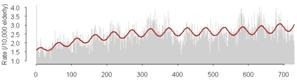				
CABELL CO, WV	2.90 (2.69, 3.11) * ~3rd week of January	1.21	2.66 (1.75, 3.57) * ~3rd week of January	1.20
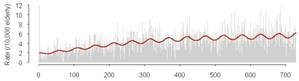				
HAMILTON CO, OH	8.93 (8.61, 9.24) * ~4th week of February	1.20	8.85 (8.28, 9.43) * ~4th week of February	1.19
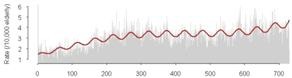				
JEFFERSON CO, KY	6.36 (6.02, 6.70) * ~2nd week of February	1.22	6.02 (4.03, 8.01) * ~2nd week of February	1.20
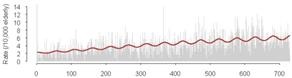				
DAVIESS CO, KY	3.39 (3.15, 3.63) * ~3rd week of January	1.20	3.19 (1.68, 4.69) * ~3rd week of January	1.20
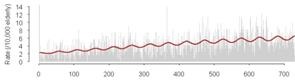				
VANDERBURGH CO, IN	7.90 (7.38, 8.41) ~3rd week of February	1.11	7.55 (6.57, 8.53) ~3rd week of February	1.11
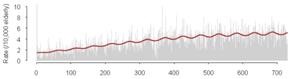				

**^†^** Adjustments were based on weekly average streamflow.

**Table 5:** Seasonal pattern for Ill-defined GI infections over 14 year-period for counties along the Ohio River with estimated peak timing and relative intensity both with and without adjustment for streamflow. Counties are listed in order based on the flow of the river, east to west. Significant (*p* < 0.05) seasonal peak estimates are denoted with an asterisk (*).

	WITHOUT ADJUSTMENT		ADJUSTING FOR STREAMFLOW ^†^	
COUNTY AND SEASONAL PATTERN	PEAK ESTIMATE (95% CI)	RELATIVE INTENSITY	PEAK ESTIMATE (95% CI)	RELATIVE INTENSITY
ALLEGHENY CO, PA	6.40 (5.97, 6.83) * ~2nd week of February	2.44	6.52 (4.93, 8.11) * ~2nd week of February	2.54
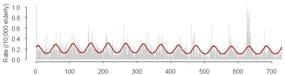				
CABELL CO, WV	5.08 (4.64, 5.51) * ~1st week of February	2.62	4.93 (3.97, 5.89) * ~1st week of February	2.56
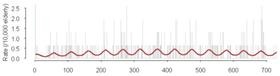				
HAMILTON CO, OH	6.88 (6.32, 7.44) ~2nd week of February	1.76	6.69 (6.29, 7.09) ~2nd week of February	1.74
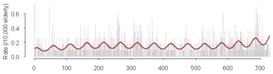				
JEFFERSON CO, KY	4.96 (4.52, 5.40) * ~1st week of February	2.57	5.52 (4.16, 6.87) * ~1st week of February	2.80
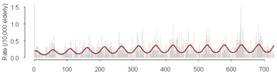				
DAVIESS CO, KY	3.69 (3.27, 4.10) * ~3rd week of January	3.88	3.79 (2.84, 4.74) * ~3rd week of January	3.94
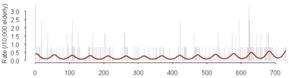				
VANDERBURGH CO, IN	3.79 (3.51, 4.08) * ~3rd week of January	2.78	4.28 (3.90, 4.67) * ~3rd week of January	2.86
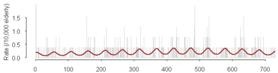				

**^†^** Adjustments were based on weekly average streamflow.

**Table 6:** Seasonal pattern for All GI infections over 14 year-period for counties along the Ohio River with estimated peak timing and relative intensity both with and without adjustment for streamflow. Counties are listed in order based on the flow of the river, east to west. Significant (*p* < 0.05) seasonal peak estimates are denoted with an asterisk (*).

	WITHOUT ADJUSTMENT		ADJUSTING FOR STREAMFLOW ^†^	
COUNTY AND SEASONAL PATTERN	PEAK ESTIMATE (95% CI)	RELATIVE INTENSITY	PEAK ESTIMATE (95% CI)	RELATIVE INTENSITY
ALLEGHENY CO, PA	8.04 (7.36, 8.71) * ~3rd week of February	1.63	8.10 (5.87, 10.33) * ~3rd week of February	1.75
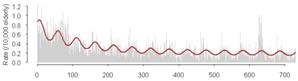				
CABELL CO, WV	4.79 (4.70, 4.89) * ~1st week of February	1.90	4.69 (3.45, 5.92) * ~1st week of February	1.87
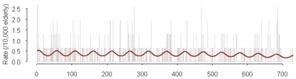				
HAMILTON CO, OH	9.38 (8.24, 10.51) ~4th week of February	1.44	9.39 (8.61, 10.17) ~4th week of February	1.44
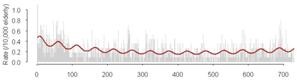				
JEFFERSON CO, KY	5.67 (5.49, 5.85) * ~1st week of February	1.82	6.47 (4.60, 8.33) * ~2nd week of February	2.04
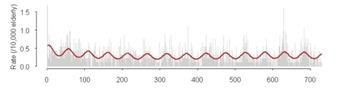				
DAVIESS CO, KY	3.13 (2.99, 3.27) * ~3rd week of January	2.61	3.16 (2.03, 4.29) * ~3rd week of January	2.61
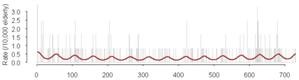				
VANDERBURGH CO, IN	4.02 (3.55, 4.49) * ~4th week of January	1.76	5.00 (4.49, 5.51) * ~1st week of February	1.84
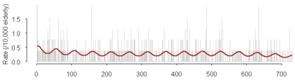				

**^†^** Adjustments were based on weekly average streamflow.

## 4. Discussion

Rivers have distinct characteristics which can affect the rate and seasonal pattern of waterborne diseases. In this paper, we compare the seasonal patterns of streamflow and various outcomes of gastrointestinal illness along the Ohio River. We demonstrated that both GI illnesses and streamflow exhibit strong seasonal patterns in the selected counties. For all outcomes the peak timing of disease preceded the peak timing seen in streamflow. We also demonstrate that the peak timing of GI illnesses did not change after adjustment for streamflow. This is the first study of its kind to address the seasonal patterns of disease outcomes along a river accounting for streamflow. Previous studies have primarily focused on one study location and measured the hydrological parameters of interest as part of the study itself [23] or had data from the water utility [21,22]. In this study we consider the entire river and utilize publicly available data to assess seasonal relationships. 

The calculated rates of gastrointestinal illness in the elderly were similar to those seen in other studies [38,39,40]. It has been demonstrated that rural private supplies have a higher risk of contamination compared to public water supplies [41]. Our findings also demonstrated the highest rates for all gastrointestinal outcomes in the one of the least populated counties selected, Cabell Co, WV. 

Our finding of peak timing of GI illness preceding the peak timing in streamflow may be because the level of pathogens in the water is diluted due to increased streamflow. Pathogens are thought to be flushed by rainfall and resulting runoff into surface waters [27,42]; however, research has demonstrated that the pathogen concentrations are lower during periods of higher streamflow due to dilution [43]. We did not observe this dilution effect when we considered the lagged cross-correlations up to six months. Alternatively, it is possible that pathogen concentration is higher preceding peak streamflow. Data on pathogen concentrations have demonstrated peak concentrations in the spring prior to peak streamflow [5]. The first flush phenomenon suggests this is possible as the majority, up to 90 percent, of pollutants are carried in the first 25 mm of runoff from a storm [44]. The data on gastrointestinal outbreaks is consistent with the concept of a first flush of pathogens and an increased likelihood of pathogens passing through treatment facilities as the majority of outbreaks are seen after severe precipitation events [31].

Additionally, pathogen concentrations are dependent on human and, to a degree, animal disease which demonstrate seasonal patterns as well [45]. For example, cryptosporidiosis concentrations are higher on the land during the spring months due to increased fecal contamination from new born calves which have higher concentrations of the pathogen [46]. Studies have also demonstrated a surge of pathogens with early snowmelt and runoff at the beginning of the rainy season with decreasing concentrations through the rainy season [10,47]. These variations in land use and topography may help explain our finding of a late peak for GI symptoms in the upper-most river site of Allegheny Co, PA. Future analyses will need to account for additional parameters which can affect runoff into surface waters, such as land use and land cover. 

For this analysis, we used broad outcome categories which do not specify a particular waterborne pathogen therefore we may not have been able to detect a relationship between peak timing of streamflow and GI illness. Since testing for specific pathogens is not commonly practiced [36,48], reporting for these diseases is limited. Marjowicz *et al*., for example, estimated that for each case in Ontario, Canada of a reportable gastrointestinal disease, there were between 105 and 1,389 cases (median 285) which went unreported, primarily because of a lack of testing [49]. With low counts of hospitalizations, we were not able to use pathogen specific outcomes and used broader outcome categories for seasonality assessment. These broad outcome categories include pathogens which have different incubation periods and seasonal patterns. The incubation period for viruses, such as norovirus and rotavirus, is only 24–28 hours whereas, the incubation period for protozoa, such as *Cryptosporidium* and *Giardia*, is typically much longer, averaging 7 days. Therefore, using these broad outcome categories that include multiple pathogens can mask the relationship with streamflow. Additionally, these different pathogens demonstrate differing seasonal patterns. Viral infections typically demonstrate higher incidence in colder, drier times of the year [50,51,52]. Our recent findings also demonstrate that the broad outcome categories for GI infections may be dominated by untested viral infections and therefore demonstrate peaks at colder times of the year [48]. A limitation of this study is that we are not able to tease apart GI illness due to non-waterborne transmission, such as food borne transmission. Future research should consider alternative modeling methods, such as a zero-inflated Poisson distribution, to assess seasonal patterns of the more specific disease outcomes with low counts. 

Another limitation of the study was the lack of availability of hydrological data. Although the USGS has monitoring stations located throughout the United States the data collection has been irregular and sporadic over time. We considered examining several water quality parameters, such as turbidity, for this analysis however, only streamflow data were available for all counties of interest for the entire time period. While streamflow is highly correlated with water quality parameters [24] including turbidity, it is not a direct measure of microbiological water quality. Available streamflow data were not gathered from main Ohio River; but for four of the six counties only data from tributaries were available. Under the Clean Water Act, states are required to ensure that their water use for public water supplies protects the fish, wildlife and recreational uses of the water bodies [53]. Under this broad mandate, states regulate and monitor streamflow levels [53] which is why this parameter was the only one available for all counties for the length of this study. The local water utilities are required to monitor finished water supplies for water quality however; they are only required to report violations to the public and the Environmental Protection Agency (EPA). Therefore, in order to consider other hydrological parameters, such as turbidity, in this type of a long term time-series study would require the cooperation of local water utilities for all locations of interest. To compound the problem, government funding for monitoring of health and environmental measures has been reduced consistently over recent years and therefore, data collection has been reduced in almost all government agencies [54]. 

We selected counties with population centers (*i.e*., large cities) which are in close proximity to the Ohio River as it was assumed they are more likely to use surface waters for public water supplies, as rural towns typically use ground water. However, upon further investigation, we found that the selected counties do not rely completely on surface water sources. We gathered water source information for each county and, according to information provided by the municipalities, most of the counties use surface or mixed (both ground and surface water) water supplies (5 of 6 counties along the Ohio River). Previous studies have shown varying seasonal patterns and rates for GI illnesses by water source [14] suggesting that the pathogen transmission and/or concentrations differ by water source. However, when assessing 548 reported gastrointestinal outbreaks researchers found a similar relationship between gastrointestinal illness and rainfall regardless of water source [31]. This finding is expected as disease transmission from within one population center to adjoining ones does not stop based on political boundary. Additionally, it has been established that one community can act as the initial site of infection with secondary transmission to adjacent communities and that community level characteristics, such as socio-demographic factors, are associated with rates of gastrointestinal illness [40]. Future research should strengthen this model by adjusting for community level characteristics, such type of water supply, percent of elderly population, and socio-demographic variables. The strength of our study is that utilizing publically available data, we are able to see a consistent pattern across all six counties when it would be expected that our results would be biased towards the null. Further interdisciplinary research is required to understand the transit time and residence time for various pathogens within ground water and surface water systems and how this can impact the seasonal patterns of the pathogens and the resulting GI illness. Rigorous modeling of streamflow, which capitalizes on advancements in hydrology modeling, will greatly add to the understanding of the associations between streamflow and gastrointestinal illness.

## 5. Conclusions

In this paper, we assess seasonal patterns of gastrointestinal illnesses in the elderly along the Ohio River and compare peak timing of illness to the peak timing of streamflow. We demonstrate that after adjusting for streamflow, the seasonal patterns of GI illness remain consistent. The peak timing of GI illness systematically precedes the peak timing of streamflow. Few studies have been conducted on the association between seasonality of hydrological parameters and GI illnesses. In the United States, hydrological data are incomplete for long time periods which limited these analyses. Given the limited availability of hydrological data, a study utilizing modeled hydrological parameters would allow for better understanding of the seasonal association in streamflow and gastrointestinal illness. Further research should investigate the timing of the first flush of runoff and associations with peak timing in GI illness since the first flush is expected to carry the majority of pathogens. Our findings suggest that pathogen loading from the land or other sources into watersheds precedes the time of peak flow resulting in the earlier peak in GI illness. However, it is necessary to conduct more interdisciplinary research to fully understand the hydrological associations with seasonal patterns of waterborne disease. 

## Figures and Tables

**Figure A:**
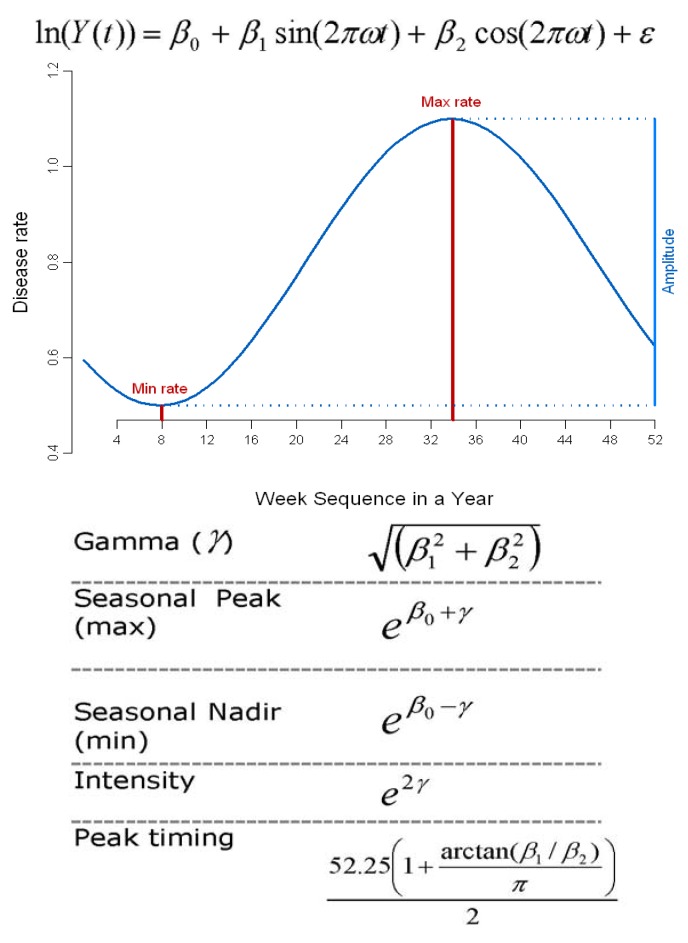
Details of the derivation of the seasonal curve properties based on the values predicted by the Poisson harmonic regression.

## Supplemental Information

**Table A:** Spearman cross-correlations of weekly time series of streamflow by county.

	Allegheny Co, PA	Cabell Co, WV	Hamilton Co, OH	Jefferson Co, KY	Daviess Co, KY	Vanderburgh Co, IN
**Allegheny Co, PA**	1.000	0.645 *	0.619 *	0.819 *	0.812 *	0.556 *
**Cabell Co, WV**		1.000	0.689 *	0.853 *	0.846 *	0.529 *
**Hamilton Co, OH**			1.000	0.821 *	0.824 *	0.708 *
**Jefferson Co, KY**				1.000	0.994 *	0.681 *
**Daviess Co, KY**					1.000	0.697 *
**Vanderburgh Co, IN**						1.000

* Correlation is significant at the 0.01 level (2-tailed).
